# GBS-SBG - GBS Serotyping by Genome Sequencing

**DOI:** 10.1099/mgen.0.000688

**Published:** 2021-12-09

**Authors:** Suma Tiruvayipati, Wen Ying Tang, Timothy M. S. Barkham, Swaine L. Chen

**Affiliations:** ^1^​ Infectious Diseases Translational Research Programme, Department of Medicine, Yong Loo Lin School of Medicine, National University of Singapore, Singapore; ^2^​ Department of Laboratory Medicine, Tan Tock Seng Hospital, Singapore; ^3^​ Laboratory of Bacterial Genomics, Genome Institute of Singapore, 60 Biopolis Street, Genome #02-01, Singapore 138672

**Keywords:** Group B *Streptococcus *serotypes, genome sequencing, short read typing, molecular epidemiology

## Abstract

Group B *Streptococcus* (GBS; *Streptococcus agalactiae *) is the most common cause of neonatal meningitis and a rising cause of sepsis in adults. Recently, it has also been shown to cause foodborne disease. As with many other bacteria, the polysaccharide capsule of GBS is antigenic, enabling its use for strain serotyping. Recent advances in DNA sequencing have made sequence-based typing attractive (as has been implemented for several other bacteria, including *Escherichia coli *, *Klebsiella pneumoniae* species complex, *Streptococcus pyogenes *, and others). For GBS, existing WGS-based serotyping systems do not provide complete coverage of all known GBS serotypes (specifically including subtypes of serotype III), and none are simultaneously compatible with the two most common data types, raw short reads and assembled sequences. Here, we create a serotyping database (GBS-SBG, GBS Serotyping by Genome Sequencing), with associated scripts and running instructions, that can be used to call all currently described GBS serotypes, including subtypes of serotype III, using both direct short-read- and assembly-based typing. We achieved higher concordance using GBS-SBG on a previously reported data set of 790 strains. We further validated GBS-SBG on a new set of 572 strains, achieving 99.8% concordance with PCR-based molecular serotyping using either short-read- or assembly-based typing. The GBS-SBG package is publicly available and will hopefully accelerate and simplify serotyping by sequencing for GBS.

## Data Summary

1. The GBS-SBG package is open source and available at Github under the MIT license (URL - https://github.com/swainechen/GBS-SBG).
2. Accession numbers of the sequencing reads and reference sequences used in the study from earlier reports have been provided within the article and in the supplementary data found at https://doi.org/10.6084/m9.figshare.16578620.v1[1]
3. The WGS data for the 572 isolates used in the study are available at https://www.ncbi.nlm.nih.gov/bioproject/PRJNA293392

**Impact Statement**

Serotyping of Group B *Streptococcus* (GBS; *Streptococcus agalactiae *) remains an important epidemiological tool for this common cause of neonatal meningitis, sepsis in adults, and foodborne illness. We have improved upon previous work to create a database and running software for serotyping GBS by whole-genome sequencing (WGS). Our new solution, termed GBS-SBG, provides full coverage of all currently described serotypes and subtypes of GBS and provides high concordance with experimental serotyping when used with two of the most common WGS data types. The new database and software should therefore be compatible with most existing WGS-based pipelines, which are becoming the standard method to type GBS (in addition to other bacteria) for epidemiological purposes.

## Introduction’

Group B *Streptococcus* (GBS, also known as *Streptococcus agalactiae *) was named for its common association with mastitis in cows (agalactiae = ‘no milk’). It is a common coloniser of the human gastrointestinal and urinary tract, being present in up to one-third of apparently healthy individuals [2–4]. Since the late 19th century, it has become increasingly associated with neonatal meningitis, today representing the most common cause [5–8]. Neonatal GBS infections are classified clinically into early-onset (<7 days of age, EOD) and late-onset (>7 days of age, LOD) disease. In addition, GBS is an increasingly common pathogen in immunocompromised and elderly adults [9–12]. Recently, GBS has been shown to cause foodborne infection associated with the consumption of raw fish in otherwise healthy adults in Singapore (and likely throughout Southeast Asia) [13–15]. Accordingly, GBS is also well known to colonize and infect (often resulting in severe invasive disease) multiple other species, including fish (where it has a large impact on aquaculture) as well as other mammals, amphibians, and reptiles [16–21]. As an important pathogen of humans, cows, and fish, GBS is therefore of concern for public health, economic, and zoonotic reasons.

Several decades ago, a serotyping system was established [22] based on differences in antigenicity of the polysaccharide capsule. Currently, there are ten main serotypes (Ia, Ib, II-IX); furthermore, serotype III has been subtyped into four subtypes (III-1 through III-4) [23]. These serotypes are encoded by the *cps* locus, which consists of both conserved (*cpsD - cpsG*) and variable (*cpsG - cpsK*) regions; the sequence differences, primarily in the variable region, form the basis of a commonly-used polymerase chain reaction (PCR) based molecular serotyping scheme [24]. The subtypes of serotype III differ in SNPs in a portion of the conserved region of the *cps* locus [25], which typically requires PCR or whole-genome sequencing to elucidate differences in subtypes III-1 to III-4 [25–27]. Epidemiological studies have made clear that different serotype (and subtype) distributions are associated with different host species, disease states, and geographical distributions [28]. For example, mastitis in cows is mostly caused by serotype Ia GBS in China [29], while serotype III (subtype III-3) is most common among cows in Canada [30]. Furthermore, outbreaks in infected fish are largely caused by serotype Ia, Ib, and III (the latter specifically being subtype III-4 and occurring predominantly in Southeast Asia, with recent introductions into Brazil) [15, 31]. These examples further highlight the epidemiological value of subtyping serotype III isolates; in particular, the subtypes of serotype III are associated with different STs and diseases in humans as well, with serotype III-4 notably associated with ST283 strains causing invasive diseases in humans and fish in Southeast Asia [15, 26, 27]. Additional resolution afforded by other typing systems (multilocus sequence typing (MLST), virulence gene typing, mobile genetic elements and antibiotic resistance profiles WGS) have overall confirmed these initial epidemiological observations based on serotypes [20, 26–28, 32–35]. Therefore, serotyping for GBS is still a useful and important method, and continues to be particularly relevant for vaccine development [28, 36].

Various experimental methods have been used for serotyping of bacteria, such as enzyme immunoassay, immunoprecipitation, co-agglutination, inhibition enzyme-linked immunosorbent assay, latex agglutination, and fluorescence microscopy [22, 37–41]. These traditional methods have mostly been replaced by genotyping methods, often based on PCR [42–45]. Given recent advances in the throughput, availability, and affordability of sequencing technologies, WGS has now become a practical method (with advantages for automation and scale) to call serotypes in multiple bacteria, such as *Salmonella* [46, 47], *Streptococcus pneumoniae* [48], *Escherichia coli* [49–51], and *Klebsiella pneumoniae* [52].

To date, three reports have explored using WGS to serotype GBS. The first used a database of nine serotypes, focusing on the variable region of the *cps* locus (*cpsG - cpsK*); this database did not include serotype IX [53] and was designed for use with assembled genomes. The second database included all of the ten main serotypes, including the full *cps* locus with both conserved and variable regions [54]. This latter database was tested using two types of workflows: (i) mapping of raw short reads, which allows direct analysis of the FASTQ files generated by all current short read sequencers; and (ii) blast of assembled sequences, which is not dependent on any particular sequencing technology; the mapping strategy was found to have higher concordance with latex agglutination-based serotyping [54]. The final mapping strategy from this report does not seem to have been implemented in any publicly available software. A third WGS serotyping strategy employed partial gene sequences of the *cps* locus as a reference database for a short-read mapping strategy [55]; the use of short (100–300 bp) partial gene sequences makes it ill-suited for analysis using an assembly-based strategy. Importantly, none of these databases included subtypes of serotype III.

We thus aimed to devise a serotyping solution using WGS for GBS that would (i) include all existing serotypes, including subtypes of serotype III and (ii) enable accurate serotyping by both short-read-mapping and assembly-based strategies. Our software is called GBS-SBG and is available at https://githubcom/swainechen/GBS-SBG.

## Methods

### Previously published GBS capsular locus reference sequences

**Fig. 1.**
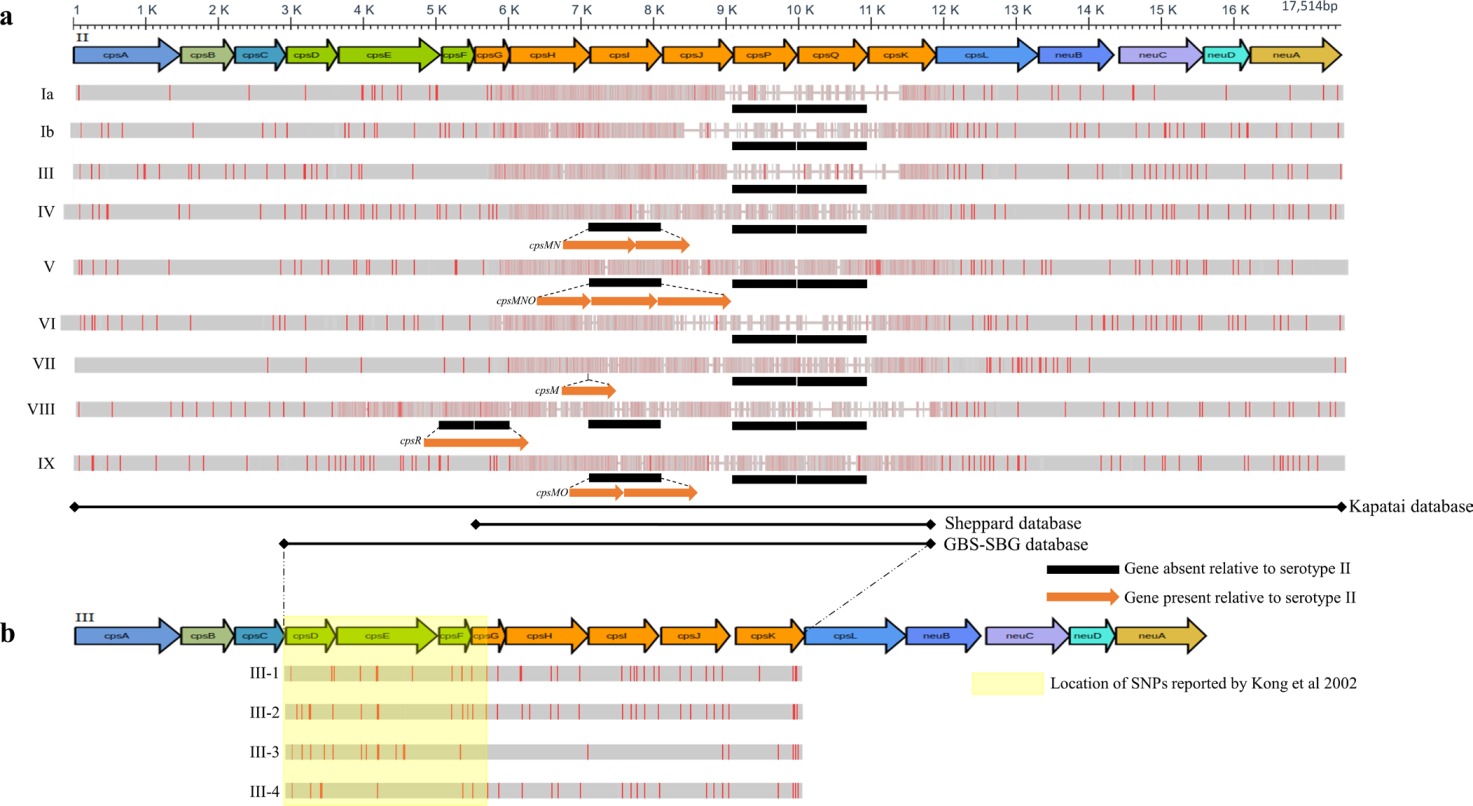
Genetic organization of the GBS *cps* locus and serotyping database strategies. (**a**) Coloured arrows at the top depict the genetic organization of the serotype II sequence. Arrows are drawn to scale, as indicated by the axis at the top. Alignments for the other nine serotype sequences (indicated by Roman numerals on the left) are shown in grey bars below. Vertical red lines indicate single nucleotide polymorphisms relative to the serotype II sequence. Black bars indicate genes that are absent from a given serotype. Orange arrows indicate genes that are unique to a given serotype, and dotted lines indicate where they fall relative to the serotype II reference. All sequences used for this figure panel are taken from Kapatai *et al*. [54]. The black lines demarcated by diamonds at each end at the bottom of the panel indicate the region of the *cps* locus spanned by the serotype sequences in the Sheppard database, Kapatai database, and GBS-SBG databases, as indicated to the right of each line. (**b**) Coloured arrows depict the genetic organization of the serotype III sequence from Kapatai *et al.* 2017 [54], using the same scale bar as for (**a**). Dotted lines between the panels show the relative alignment of the serotype II and serotype III reference sequences. Grey bars and vertical red lines indicate alignment and SNPs for the serotype III subtypes (indicated on the left). The yellow box indicates the region in which SNPs reported by Kong *et al*. [23] are located.

Reference sequences representing the ten serotypes (Ia – IX) were taken from [54]; we refer to these as the ‘Kapatai database’ (Fig. 1, Table 1). The sequences in the Kapatai database range in length from 15090 to 17514 bp. Reference sequences for nine serotypes (Ia – VIII) with lengths ranging from 4477 to 6307 bp were obtained from [53]. We refer to these nine sequences as the ‘Sheppard database’. Sequences representing two subtypes of serotype III (III-2 (AF332896) and III-3 (AF332897)) were obtained from [23]. An additional method was published in [55], though we did not test this database; we refer to this database as the ‘Metcalf database’.

**Table 1.** GBS serotype reference databases used in the current study

Serotype	Kapatai *et al*. 2017 [54]			Metcalf *et al*. 2017 [55]						Sheppard *et al*. 2016 [53]						Current study					
	Accession	L(bp)	Region	Accession	L(bp)	Start	End	Id%	Region	Accession	L(bp)	Start	End	Id%	Region	Accession	L(bp)	Start	End	Id%	Region
Ia	LT671983	15 799	***cps locus***	AB028896.2	143	6284	6426	94.41	***cpsH***	AB028896.2	4714	5538	10 254	99.15	***cpsG - cpsK***	–	6712	3539	10 250	100	***cpsD - cpsK***
Ib	LT671984	15 807		AB050723.1	143	6396	6538	100	***cpsH***	AB050723.1	4617	5650	10 266	100		–	6616	3651	10 266	100	
II	LT671985	17 514		AY375362.1	139	11 345	11 483	100	***cpsK***	EF990365.1	6307	5575	11 881	99.84		–	8315	3567	11 881	100	
III	LT671986	15 760		AF163833.1	172	6631	6802	99.42	***cpsH***	AF163833.1	4602	5592	10 193	99.33		n/a	–	–	–	–	
III-1	–	–		–	–	–	–	–	–	–	–	–	–	–		SG-M340*	6610	3593	10 202	99.33	
III-2	–	–		–	–	–	–	–	–	–	–	–	–	–		SG-M40*	6602	3593	10 194	99.35	
III-3	–	–		–	–	–	–	–	–	–	–	–	–	–		SG-M61*	6610	3593	10 202	99.67	
III-4	–	–		–	–	–	–	–	–	–	–	–	–	–		SG-M918*	6610	3593	10 202	99.45	
IV	LT671987	16 549		AF355776.1	222	6670	6891	100	***cpsH***	AF355776.1	5240	5731	10 971	99.98		–	7249	3723	10 971	100	
V	LT671988	17 319		AF349539.1	171	9107	9277	100	***cpsO***	AF349539.1	6148	5578	11 725	100		–	8156	3570	11 725	100	
VI	LT671989	15 847		AF337958.1	99	7385	7483	100	***cpsH***	AF337958.1	4477	5777	10 254	99.89		–	6477	3778	10 254	100	
VII	LT671990	16 473		AY376403.1	160	7535	7694	100	***cpsM***	AY376403.1	5264	5578	10 842	99.45		–	7272	3570	10 841	100	
VIII	LT671991	15 090		AY375363.1	113	8167	8279	100	***cpsJ***	AY375363.1	4370	5117	9487	99.98		–	5915	3541	9455	100	
IX	LT671992	16 440		GQ499301.1	130	8665	8794	100	***cpsO***	n/a	–	–	–	–		–	7273	3581	10 853	100	

Boxes in white - Start, End, Id%, Region relative to sequences from the Kapatai database.

Boxes in grey - Start, End, Id%, Region relative to the serotype III sequence from the Kapatai database.

*Isolate names from the current study; Id%: Percentage nucleotide identity; L(bp): Length in base pairs)

### GBS strains

A total of 572 GBS isolates were collected between November 2000 and July 2018. Of these, 547 were isolated from humans and 25 from fish. 487 of the human isolates were collected at Tan Tock Seng Hospital (TTSH), Singapore, and stored at −70 °C. All isolates were from nonpregnant adults, as TTSH does not offer paediatric or obstetric care. We obtained/collected 45 (human), six (fish), 20 (one human, 19 fish), 14 (human) isolates from Laos, Malaysia, Thailand, and Vietnam, respectively. Additional data on the isolates, including the isolation source, are included in Data S3 (available in the online version of this article).

### PCR-based molecular serotyping

We refer to the use of PCR assays to determine serotypes as ‘molecular serotyping’. GBS isolates were subcultured onto blood agar and re-identified using a MALDI-TOF system (Bruker). GBS DNA was extracted with the EasyMag system (BioMérieux) according to the manufacturer’s instructions. Molecular serotyping of the GBS isolates was performed using two PCRs: ‘Multiplex PCR 1’ and ‘Multiplex PCR 2’ as described by Poyart *et al.* [56], with segregation into capsular polysaccharide serotypes Ia, Ib, II, III, IV, V, VI, VII, and VIII achieved using agarose gel electrophoresis based on amplicon size. If an isolate remained untypeable after these two PCRs, a third PCR step with another multiplex PCR as described by Imperi *et al.* [24] was then performed.

### Whole-genome sequencing and analysis

Genomic DNA was extracted from overnight cultures on blood agar using the QIAamp DNA mini kit (cat. no. 51306), including a preliminary enzymatic lysis step and an extra centrifugation step, as follows. Colonies were harvested with a sterile swab, suspended in 500 µl nuclease free water, and pelleted at 14680 r.p.m. for 3 mins. The pellet was resuspended in 200 µl of Enzymatic lysis buffer by vortexing, then incubated at 37 °C for 1–2 h. After this incubation, 50 µl proteinase K and 250 µl buffer AL (from the QIAamp kit) were added and mixed by vortexing. These were then further incubated at 56 °C for 1 h, followed by an extra centrifugation step for 5 mins at 14680 r.p.m. Then, 420 µl of the resulting supernatant was used for DNA extraction using the QIAamp protocol. The composition of the enzymatic lysis buffer was: 20 mM Tris-HCl (pH 8.0), 2 mM sodium EDTA, 1.2 % Triton X-100, 20 mg ml^−1^ lysozyme. Whole-genome sequencing was performed by the Next Generation Sequencing Platform at the Genome Institute of Singapore, as previously described [14]. Briefly, sequencing library preparation was done with the use of the TruSeq Nano DNA LT Library Prep Kit (Illumina) or the Nextera XT Library Prep Kit (Illumina) according to the manufacturer’s instructions. The sequencing libraries were sequenced using a NextSeq 500 or HiSeq 4000 sequencer with 2×151 bp reads (Illumina, San Diego, CA, USA). All sequencing data is uploaded in GenBank under BioProject PRJNA293392. The estimated sequencing depth is included in Data S3; the mean and the range of the sequencing depth for the 572 isolates are 175.6x and 14.6–1553.8x, respectively.

SRST2 version 0.2.0 [57] was used to call MLST using reference sequences downloaded from PubMLST (https://www.pubmlst.org). SRST2 was also used to call serotypes using a mapping strategy using the GBS-SBG database as a custom reference database, according to the SRST2 documentation. The default minimum percentage coverage cutoff (--min-coverage option; default 90%) for gene reporting was used.

Raw short read (FASTQ) sequences were also assembled with velvet (version-1.2.10) [58] using the VelvetOptimizer helper script (version 2.2.4) and a minimum contig cutoff of 500 bp, scaffolded with OPERA-LG (version 2.0.6) [59], and finished with FinIS (version 0.3) [60]. Alignment-based calling of serotypes was done using a single script that performed alignment against the GBS-SBG database using blastn [61] and processed the results (available at https://github.com/swainechen/GBS-SBG). For consistency with SRST2, a minimum nucleotide identity of 90% across 90% of the reference serotype length (i.e. 90% coverage) was required for making a serotype call.

### Creation of the GBS-SBG database

For all serotypes except for serotype III, the sequences from the Kapatai database [54] were used (Table 1). Multiple sequence alignments were made using MAFFT version 7.45 [62]. Sequence alignments were visualized using Jalview 2.11.1.3 [63]. Sequences were trimmed to a uniform 5′ and 3′ end (see results) (Fig. 1). For the subtypes of serotype III, reference sequences for III-2 and III-3 were taken from [23]. SNPs specific for III-1 and III-4 [23] were introduced into the reference serotype III sequence from the Kapatai database [54] (of note, this serotype III sequence (LT671986) appeared to be a hybrid between III-2 and III-4 (based on characteristic SNPs for these subtypes) and was therefore not clearly subtypeable itself (Data S1)). To generate reference sequences for III-1 and III-4, all of the new strains that were typed as serotype III by PCR (173/572 strains) were considered. All of these were unambiguously typeable as one of the four subtypes of serotype III based on the SNPs reported by [23] (Data S1, Fig. S1). For each subtype, one corresponding assembled sequence (Table 1) was then included as a reference sequence in the final database.

### Pairwise distance and phylogenetic analysis

Pairwise distances and maximum likelihood phylogenetic analysis using a Tamura-Nei model [64] was performed using mega X [65]. The initial tree(s) for the heuristic search were obtained automatically by applying the Neighbour-Join and BioNJ algorithms to a matrix of pairwise distances estimated using the Maximum Composite Likelihood (MCL).

## Results

### Existing GBS serotyping strategies do not include all known serotypes and only work with a single data type

A valuable dataset, comprising 790 isolates with WGS data and experimental serotyping by latex agglutination, was made publicly available in the report of the Kapatai database, one of the short-read mapping GBS serotypers [54]. This study achieved a concordance of 725/790 between WGS (using short read mapping) and latex agglutination. The authors also tested an assembly-based method using their same sequence database, but found lower concordance (664/790) (Data S2). The lower concordance using assemblies, of course, meant that the concordance between the mapping- and assembly-based strategies was also not perfect (721/790).

We performed serotyping of these 790 isolates using a short read mapping strategy (with the popular SRST2 programme) against the Kapatai database; this resulted in 431/790 correct serotypes. The 358 discrepant calls were all miscalls between serotypes III and Ia (355 were miscalled as Ia while three were miscalled as III). Of note, high similarity between the *cps* loci for serotypes Ia and III, leading to a potential for miscalls, has previously been reported [23, 54, 66].

We further leveraged this data set of 790 strains to evaluate the suitability of the Sheppard database [53] (designed for an assembly-based strategy) using a mapping-based strategy; this performed very well, resulting in 780/790 correct serotype calls. Of the ten discordant calls, nine were serotype IX (which was not present in the Sheppard database) while one was miscalled as serotype III (instead of Ia).

The Metcalf database [55] was also originally designed for mapping-based typing. We did not assess its performance for assembled sequences because the reference sequences were very short (100–300 bp) (Table 1).

Therefore, none of the three existing databases was definitively usable as-is for both mapping- and assembly-based typing. Furthermore, none included the possibility of calling serotype III subtypes.

### Construction of a complete reference serotype database usable for both assembly- and short read-based analyses

We therefore sought to construct a single database that could provide high accuracy using both short reads and assemblies as well as provide serotype III subtyping. Previous studies have noted that the variable region of the *cps* locus is important for assembly-based typing; this was the main region used by the Sheppard database with an assembly-based method [53]. Short read mapping-based typing, such as that performed by SRST2 (which was used with the Metcalf database [55]), is typically designed to differentiate between closely related alleles (i.e. conserved regions), making it well suited for MLST, for which the 5′ and 3′ ends of the reference sequences are strictly trimmed to provide uniform-length alignments of the typing alleles. Interestingly, we found that using SRST2 also worked well with the Sheppard database but less well with the Kapatai database, despite the former including only the variable region of the *cps* locus.

We therefore hypothesized that a new database should include both conserved and variable portions of the *cps* locus, in order to accommodate both short read- and assembly-based typing. We also sought to align the start and end of each sequence. We built upon the Kapatai database [54], as it already achieved good performance with short read mapping (though not with SRST2).

We constructed a new database as follows (see Methods for more details):

1. Take all ten main serotypes from the Kapatai database and align them.
2. Trim all sequences on the 5′ end to the region that includes the SNPs reported to differentiate serotype III subtypes [23].
3. Trim all sequences on the 3′ end to the edge of the variable sequence, which corresponds to the right edge of the sequences in the Sheppard database; this resulted in an ~8 kbp reference sequence for each serotype.
4. Replace the single serotype III sequence with representatives from the four subtypes (see below).

The serotype III subtypes have been defined by SNPs that are found in a conserved region of the *cps* locus [23]. Full length sequences for these have not been explicitly identified, however. We therefore used the WGS assemblies for our newly sequenced strains (see below) that were typed as serotype III by PCR (173 strains in total). These were unambiguously classifiable into the four subtypes based on the SNPs reported by [23], resulting in 26 III-1, 22 III-2, eight III-3, and 117 III-4 isolates. For each subtype, we then took the consensus sequence over the ~8 kbp typing region (for each subtype, this consensus sequence exactly matched the sequence of multiple assemblies in our data set) and included this in our new reference serotyping database. We refer to this as the GBS-SBG database.

### The GBS-SBG database enables serotyping by both mapping and assembly-based strategies

We tested the GBS-SBG database on the 790 isolates from Kapatai *et al.* [54], using both short read- (with SRST2) and assembly-based (using blastn, with 90% identity and 90% coverage cutoffs; see Methods) strategies. We used the mapping-based calls from Data S2 in the original report [54] as the gold standard (as noted above, these mapping-based calls were only concordant with assembly-based calls for 721/790 strains in the original report). We found 100% concordance (790/790) and 94.6% (748/790) with the reported serotypes when using read mapping (SRST2) and assembled sequences (blastn), respectively, with the GBS-SBG database. The differences in the 42 strains from the assembly-based analysis were all due to calls as non-typeable. Further examination showed that these 42 strains had lower sequencing depth (*P*<1.044e-12, two-tailed Mann-Whitney U-test; Fig. S4), which led to an inability to assemble portions of the *cps* locus. The regions that were not assembled correlated with areas of low coverage, which still largely remained above the minimum read depth (5x) required by SRST2 to make a call (Fig. S5). Use of the original reported assemblies [54], which were performed using SPAdes instead of velvet, led to a different set of nontypeable strains, which also had generally lower coverage (Data S2).

### Validation of GBS-SBG on a previously unanalyzed set of strains

A total of 572 previously unanalysed GBS isolates were serotyped by PCR and whole-genome sequenced. By PCR, none of these were typed as serotype VIII, and one isolate (SG-M122) was non-typeable (Data S3). We again used both short read- (with SRST2) and assembly-based (using blastn) strategies with the Sheppard and Kapatai databases as well as with the GBS-SBG database. Using the GBS-SBG database achieved the best concordance by both mapping- and assembly-based strategies (571/572, 99.8% concordance) (Data S3). Furthermore, we achieved 100% accuracy in calling serotype III subtypes. For assembly-based analysis only, the Sheppard database was equivalently good, with the exception of not including a serotype IX reference sequence.

**Fig. 2.**
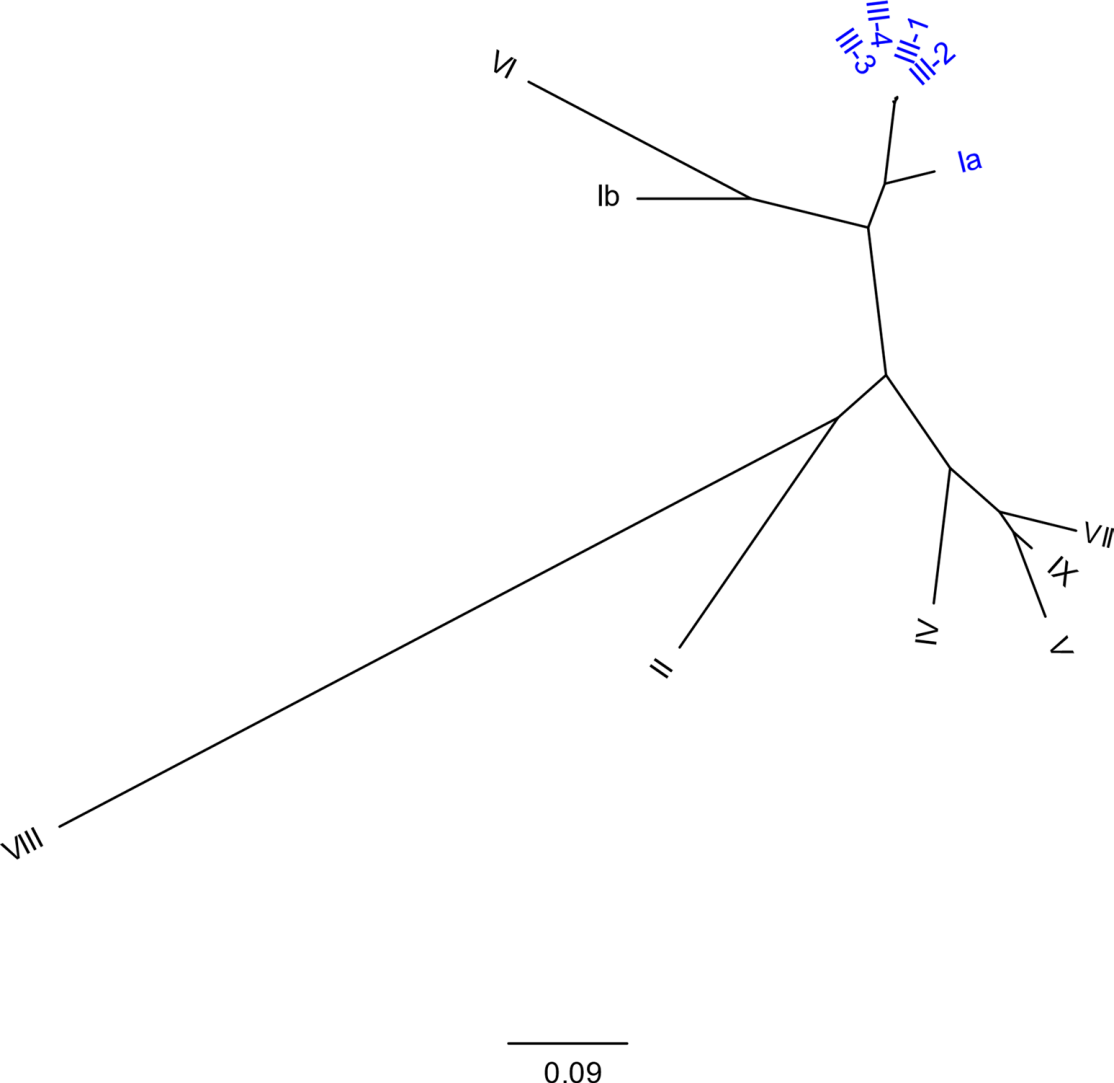
Phylogenetic relationship of serotype reference sequences in the GBS-SBG database. A maximum likelihood tree is shown; a total of 13 serotype sequences were used to plot an unrooted binary tree. The scale bar for branch length at the bottom indicates the number of substitutions per site. There were a total of 11911 positions in the alignment. The labels for serotypes Ia and subtypes of serotype III are highlighted in blue for ease of visualization.

Interestingly, using a mapping-based strategy (although with SRST2), the Kapatai database led to concordant calls with PCR serotyping in only 386/572 isolates. The majority of the discrepancies (168/186) were due to a miscall of serotype Ia (by WGS) for serotype III (by PCR). The remaining 18 isolates had the opposite problem (called serotype III by WGS using the Kapatai database and serotype Ia by PCR) (Data S3). As noted above, this is consistent with the close similarity between serotype Ia and III [23, 54, 66], which we saw with the reference sequences we used as well (Figs 2 and S2). Furthermore, the serotype III sequence included in the Kapatai database itself was not clearly one of the previously described subtypes, but instead appeared to be a hybrid of III-2 and III-4 (Data S1). These miscalls between serotypes Ia and III were resolved by using our new database, possibly in part by the inclusion of all four subtypes and by the alignment of the 5′- and 3′-ends of the reference sequence, facilitating discrimination by SRST2.

We found only one strain (SG-M666) that had a discordant call with PCR (Ia) and WGS (V) typing (both short reads and assemblies gave a serotype V call). On repeat PCR typing, this strain was again called as serotype Ia. Examination of the WGS data showed that the *cps* locus was 100% identical to the serotype V reference sequence, with a 614 bp deletion affecting the *cpsN* and *cpsO* genes. As this deletion is <10% of the length of the serotype V reference sequence, GBS-SBG called this as serotype V (Fig. S3). In contrast, the SG-M666 *cps* locus was only 98.8% identical to the serotype Ia reference sequence in regions where it was aligned by blastn. Furthermore, it had a 3423 bp deletion (>10% of the total length) relative to the serotype Ia reference sequence (such that the *cpsG-cpsJ* genes were missing). Closer examination of the target regions for the typing PCRs showed that the 614 bp deletion (relative to the serotype V reference sequence) eliminated one of the priming sites for the serotype V PCR; in this multiplex PCR, serotype Ia and serotype V have two PCR products of identical size, but serotype V should have a third PCR product that overlapped this deletion. This led to a miscall of this strain as serotype Ia by PCR (Fig. S3). Therefore, while discordant between PCR and WGS, we believe that in this case the WGS typing (serotype V) is the correct call, though the 614 bp deletion may mean this strain is actually nontypeable by latex agglutination.

## Discussion

The dramatic increase in availability of whole-genome sequencing has increased the importance of *in silico* approaches for analysis of multiple bacteria. Serotyping remains an important epidemiological adjunct for many bacteria of public health importance. For GBS, three previous studies have reported WGS-based serotyping, two using a mapping approach (one using SRST2 and another using a custom SNP-based method) [54, 55] and one using an assembly approach [53]. Having a database that is compatible with a general tool like SRST2 (as opposed to a custom mapping method) provides advantages for enabling standard analysis of multiple bacteria, such as for MLST, antibiotic resistance, and virulence genes. SRST2 also includes a reference database enabling serotyping of *E. coli *. However, many WGS-based bacterial serotyping methods (such as for *E. coli *, *K. pneumoniae *, *Salmonella *, Group A *Streptococcus *, etc.) still use an assembly-based analysis [52, 67–69]. Furthermore, long-read sequencing technologies are also becoming more popular, which likely will be better suited to assembly-based methods. We therefore speculate that both short read- and assembly-based methods will be important for some time; however, finding a common reference database which works equivalently well for mapping- and assembly-based serotype calling for GBS has been challenging [54].

Here, we have developed a single database (GBS-SBG) that provides the highest accuracy (as assessed by concordance with PCR-based molecular serotyping) for serotyping GBS, regardless of whether a mapping- or assembly-based strategy is used. This database can be used directly by the popular SRST2 programme. Previous studies had already noted that use of the full *cps* locus, the conserved *cpsD-G* genes [53], or the variable *cpsG-K* genes [54, 55] were not simultaneously suitable for use with both mapping- and assembly-based analyses. Therefore, we included portions of both the variable and conserved regions. Inclusion of a part of the conserved region enabled us to further incorporate the discriminating SNPs for subtypes of serotype III [23]. Finally, to match other databases provided with SRST2, we ensured that the reference sequences had aligned 5′- and 3′-edges.

We validated the accuracy of serotyping with this database using a previously published dataset of 790 sequenced strains (typed with latex agglutination) [54], achieving equal or higher concordance than the original report, regardless of whether a mapping- or assembly-based approach was used. Calls based on analysis of assemblies appear more sensitive to sequencing depth, with lower sequencing depth correlating with an increase in non-typeable calls. This in turn may differ based on the assembly methods used. Hence, we recommend to ensure at least ~60x average read depth, which appears to effectively eliminate the influence of the choice of assembly method. In cases where sequencing coverage is below 60x, GBS-SBG can be set to report the next best call (i.e. with a coverage <90%); these were all concordant with the mapping-based analysis and the gold standard serotypes reported by Kapatai *et al.* [54]. We further validated the accuracy with a new dataset of 572 sequenced strains (typed by PCR), achieving 99.8% (571/572) concordance using either analysis method. On careful examination, the single discordance appeared to be caused by an error in the PCR-based serotyping, due to a 614 bp deletion in an otherwise canonical serotype V locus that affected the priming sites for one of the PCRs. It remains possible that the WGS call (serotype V) is also incorrect, as the 614 bp deletion affects genes important for capsule assembly, and this strain may actually be nontypable by latex agglutination. Indeed, nontypeable strains can be caused by loss of function mutations in the *cps* genes, and this affects all GBS lineages examined [70]. Regardless, this example highlights the advantage of WGS in providing more data for serotyping and in being less susceptible to sequence variants or mutations that affect PCR priming sites, and provides a further use case for GBS-SBG to report the next best call.

We attempted to use data sets reported by the other two reported WGS serotyping methods (corresponding to the Sheppard and Metcalf databases) [53, 55]. We were unable to locate sequencing data for the strains analysed by Sheppard *et al.* [53] in public repositories. Serotype data for individual strains was not reported in [55]; however, the number of strains for each serotype was reported in aggregate, and WGS-based serotyping using our new database, with either analysis method, reproduced a similar serotype distribution (data not shown).

In conclusion, we found that use of the GBS-SBG database enables accurate WGS-based serotyping of GBS by both mapping- and assembly-based strategies. This new database provides a further advantage over previously reported databases in allowing subtyping of serotype III; use of both conserved and variable regions of the *cps* locus also provides a clear guide for potential inclusion of additional serotypes or subtypes that may be described in the future. The mapping-based strategy is compatible with the commonly used SRST2 tool. The ability to also use an assembly-based strategy with the same reference database increases the generality and anticipates a continued transition to long-read sequencing.

## Supplementary Data

Supplementary material 1

Supplementary material 2
