# Supplementary material for: GBS-SBG - GBS Serotyping by Genome Sequencing
Source: Microb Genom. 2021 Dec 9;7(12):000688. doi: 10.1099/mgen.0.000688 (PMC9842102; doi:10.1099/mgen.0.000688)

Supplementary Figure 1. Phylogenetic tree of serotype III sequences. A maximum likelihood tree is shown; a serotype Ia sequence was used as an outgroup to root the tree. This analysis involved 175 nucleotide sequences. The scale bar for branch length at the bottom indicates the number of substitutions per site. There were a total of 6789 positions in the final dataset. Branch colors indicate the subtype of serotype III, as indicated by the legend at the top left. The sequence labelled in black and highlighted with an arrow is the reference serotype III sequence from the Kapatai database.

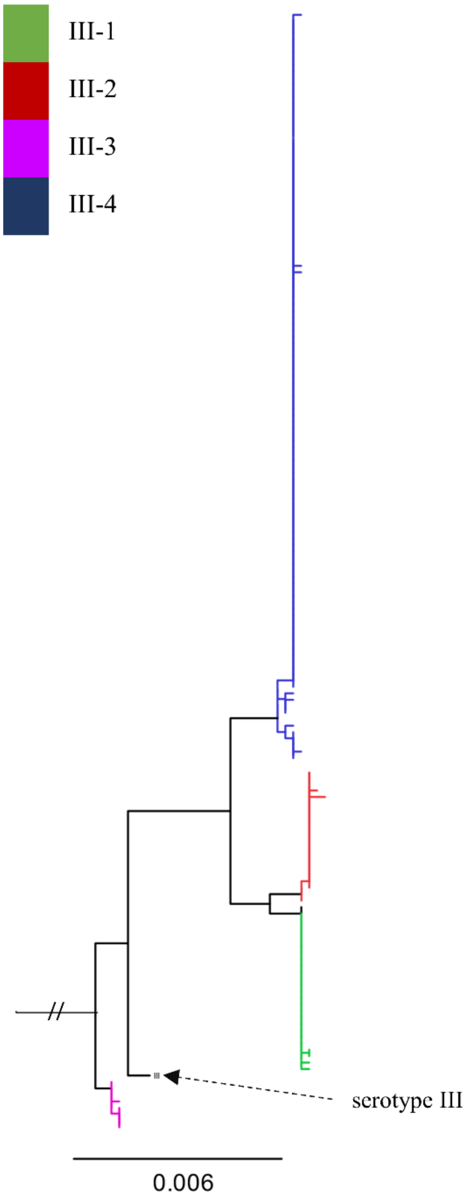

Supplementary Figure 2. Typeability based on best versus second-best serotype match. Each graph plots the total Bitscore (reported by BLASTN) for the second best-scoring serotype (y-axis) against the total Bitscore for the best-scoring serotype matched (x-axis). A solid black line is drawn at y=x; thus, points further to the right of the black line indicate better typeability (i.e. a larger difference in total Bitscore between the best and second-best match). (a) Each data point represents one strain (n = 790 total). Points are colored based on the best serotype matched, as indicated by the legend on the right. (b) Only strains with a best match to one of the serotype III subtypes are plotted (n = 356 total). Each strain is represented by two data points: one point is as plotted in panel (a), while a second data point is plotted in which the second-best match was taken to be the best non-serotype III match. Thus, the two data points for each strain are aligned vertically. Points are colored based on the serotype of the second-best match as indicated by the legend on the right.

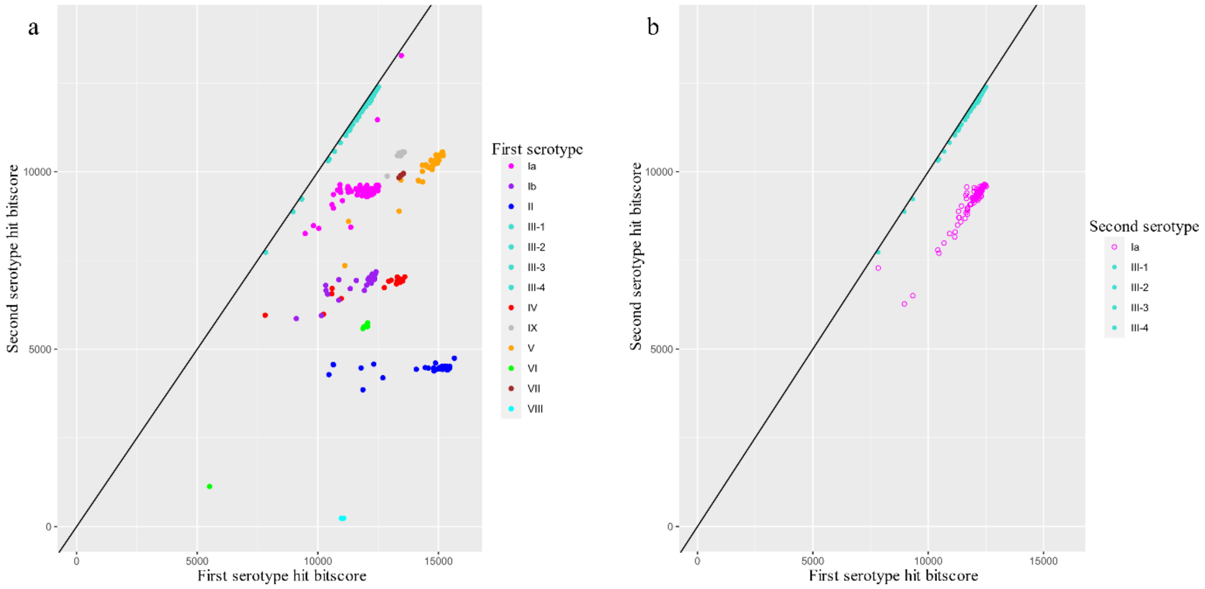

Supplementary Figure 3. Detailed analysis of PCR versus WGS serotyping of SG-M666. Colored arrows in the middle track depict the genetic organization of the reference sequences for serotype Ia and V. Colors for each gene are the same as shown in Figure 1. Genes conserved between serotype Ia and V are shown in the middle. Genes unique to serotype Ia and serotype V are shown offset above and below, respectively, the conserved genes. Dotted lines join contiguous sequences, which are offset due to different lengths of unique genes. A nucleotide scale bar for each serotype is shown (Ia above, V below). Solid colored bars adjacent to the nucleotide scale indicate the percent nucleotide identity (as reported by BLASTN) between SG-M666 and the serotype Ia and serotype V reference sequences, according to the color legend at the bottom left. The location of the PCR amplicons used for PCR-based serotyping (Poyart C, et al. 2007, Imperi M, et al. 2010) are shown as colored lines above (for serotype Ia) and below (for serotype V) the nucleotide identity tracks; those with coordinates overlapping black bars on the “% nucleotide identity” track are predicted to yield no amplification. Of the three PCRs from Imperi, et al. 2010, the PCR that distinguishes serotype V from serotype I (at ~4.6-5.2kb on the serotype V scale; highlighted with an asterisk) is predicted to be negative, thus mimicking a serotype I result.

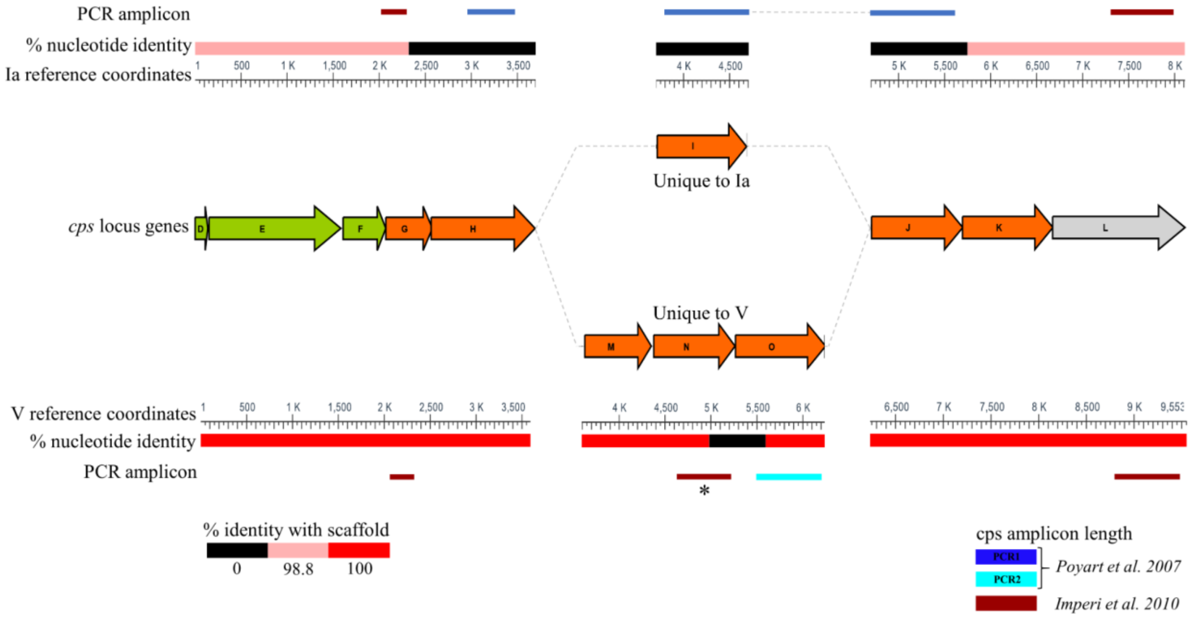

Supplementary Figure 4. Box plot showing the distribution of the average read depths in the Kapatai datasets. The median is indicated by the horizontal line in the middle of each box and the 75th and 25th percentiles indicated by the top and bottom borders of the box, respectively. Whiskers indicate 1.5x the inter-quartile range. Outliers are represented by individual data points. The Mann–Whitney U test was used to compare the median read depths of the non-typeable (*n=43*) and typeable (*n=747*) datasets (p< 1.044e-16) .

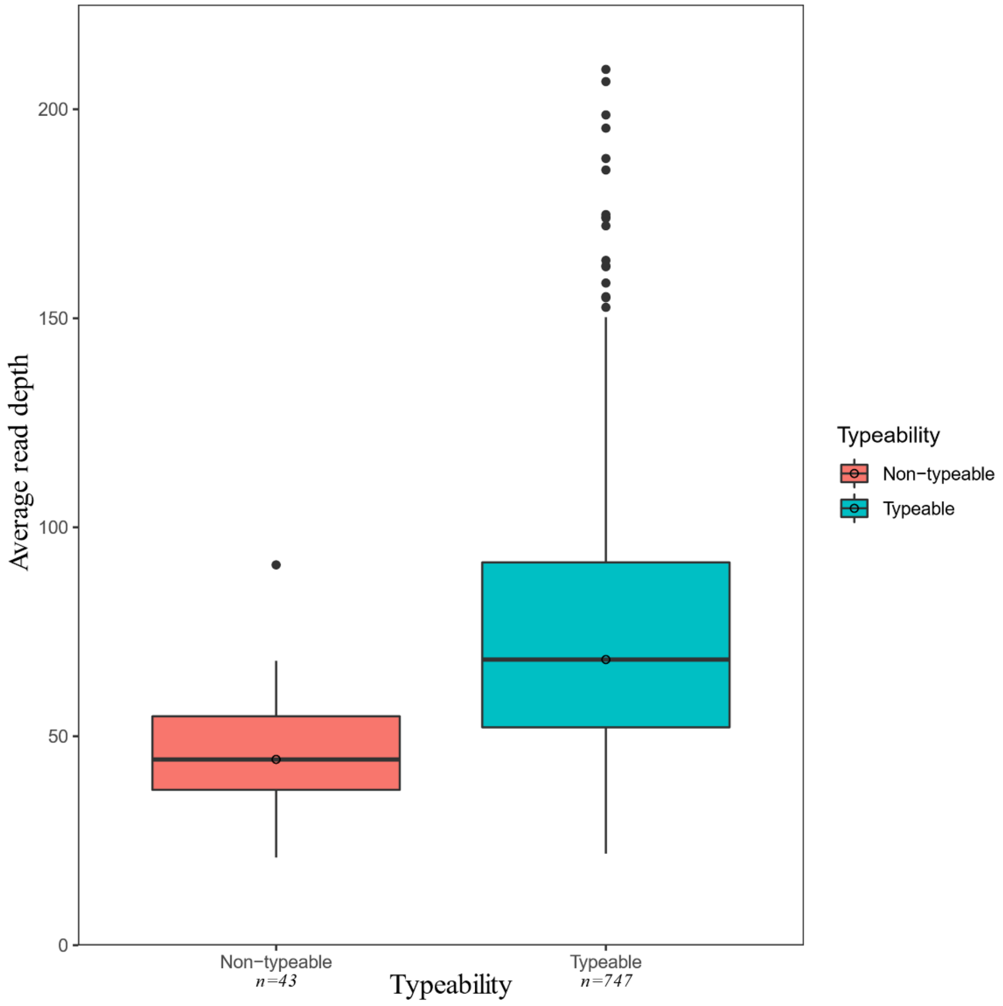

Supplementary Figure 5. Detailed analysis of a GBS-SBG non-typeable and typeable dataset. Each graph plots the read depth per reference base (y-axis) against the serotype II reference coordinate (x-axis). A solid red line is drawn at y=5, which is the minimum read depth required by SRST2 to consider a base "covered". (a) PHEGBS0096 (ERR1741774), which was typed as serotype II by SRST2 using short reads (20.967x average read depth). This was called as nontypeable by GBS-SBG using the assembly; the next best hit was serotype II, with 99.9% identity over 68.5% coverage. (b) PHEGBS0074 (ERR1742026) was typed as serotype II by both SRST2 (for short reads) (23.678x average read depth) and GBS-SBG (for assemblies). Note that the coverage does not fall as low as it does for PHEGBS0096, and the assembly of the cps locus is more complete. Solid colored bars at the bottom of each graph indicate the percent identity (as reported by BLASTN) between the serotype II reference sequence (8315bp) and PHEGBS0096 and PHEGBS0074 samples, according to the legend between the panels.

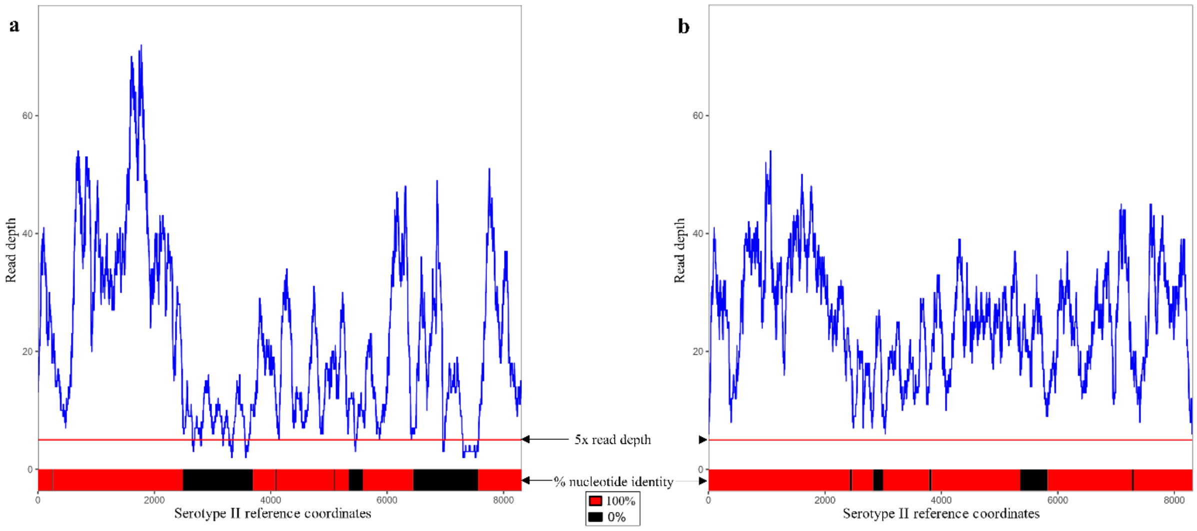

Supplement: Supplementary material 1 [file mgen-7-0688-s001.pdf]
